# Review of concepts in therapeutic decision-making in HER2-negative luminal metastatic breast cancer

**DOI:** 10.1007/s12094-019-02269-7

**Published:** 2020-02-12

**Authors:** I. Alvarez-Lopez, S. Bezares, E. Dalmau Portulas, E. García-Martínez, J. Á. García-Sáenz, M. Gil-Gil, E. Martínez de Dueñas, N. Ribelles, A. Santaballa Bertrán

**Affiliations:** 1 Hospital Universitario Donostia–BioDonostia, Begiristain Doktorea Pasealekua, 109, 20014 Donostia, Gipuzkoa Spain; 2 Grupo GEICAM de Investigación en Cáncer de Mama/GEICAM Spanish Breast Cancer Group, San Sebastián de los Reyes, Madrid, Spain; 3 Parc Taulí, Hospital Universitari, Sabadell, Barcelona, Spain; 4 Hospital General Universitario Morales Meseguer, Murcia, Spain; 5 Hospital Clínico San Carlos, Madrid, Spain; 6 Centro de Investigación Biomédica en Red de Oncología, CIBERONC-ISCIII, Madrid, Spain; 7 ICO Bellvitge, Hospitalet del Llobregat, Barcelona, Spain; 8 Consorcio Hospitalario Provincial de Castellón, Castellón de la Plana, Spain; 9 Unidad de Gestión Clínica Intercentros de Oncología Medica, Hospitales Universitarios Regional y Virgen de la Victoria de Málaga, 29010 Málaga, Spain; 10 Instituto de Investigación Biomédica de Málaga (IBIMA)-CIMES-UMA, 29010 Málaga, Spain; 11 Hospital Universitario y Politécnico La Fe, Valencia, Spain

**Keywords:** Consensus, Metastatic breast cancer, Luminal, Hormonal therapy, Hormonal receptor, Hormonal resistance

## Abstract

**Purpose:**

Hormone receptor (HR)-positive, Human epidermal growth factor receptor 2 (HER2)-negative metastatic breast cancer (MBC) requires a therapeutic approach that takes into account multiple factors, with treatment being based on anti-estrogen hormone therapy (HT). As consensus documents are valuable tools that assist in the decision-making process for establishing clinical strategies and optimize the delivery of health services, this consensus document has been created with the aim of developing recommendations on cretiera for hormone sensitivity and resistance in HER2-negative luminal MBC and facilitating clinical decision-making.

**Methods:**

This consensus document was generated using a modification of the RAND/UCLA methodology, which included the definition of the project and identification of issues of interest, a non-exhaustive systematic review of the literature, an analysis and synthesis of the scientific evidence, preparation of recommendations, and external evaluation with a panel of 64 medical oncologists specializing in breast cancer.

**Results:**

A Spanish panel of experts reached consensus on 32 of the 32 recommendations/conclusions presented in the first round and were accepted with an approval rate of 100% about definition of metastatic disease not susceptible to local curative treatment, definition of hormone sensitivity and hormone resistance in metastatic luminal disease and therapeutic decision-making.

**Conclusion:**

We have developed a consensus document with recommendations on the treatment of patients with HER2-negative luminal MBC that will help to improve therapeutic benefits.

## Introduction

Breast cancer accounts for 30% of all cancers of the female gender, and is the most common malignancy in women and the main cause of cancer death. In Spain, the age-adjusted annual incidence is 125.8 cases/100,000 inhabitants and the mortality rate is 22.7 cases/100,000 inhabitants [1]; 32,825 new cases were diagnosed in 2018 [2, 3].

Between 5 and 6% of breast cancer patients have metastatic disease at the time of diagnosis, and approximately 30% of patients diagnosed in the early stages will experience a relapse with the appearance of distant metastases [4]. Most patients diagnosed with MBC are postmenopausal women between 50 and 79 years of age (64–79%) [4]. Overall survival (OS) varies according to each biological subtype, and in the HR-positive, HER2-negative subgroup, median OS is 42 months. At present, MBC is still incurable and the main treatment goal is to improve OS and quality of life [5].

Treatment response in MBC depends mainly on the biological subtype. In the HR-positive, HER2-negative subtype, several factors play a significant role, including status at diagnosis (“de novo” or relapse after treatment of local disease), previous treatment for local disease, time to relapse, response to previous treatments for disseminated disease, time to next progression, progression-free survival (PFS), and hormone sensitivity criteria in patients with tumors that express hormone receptors. The first-line treatment recommended for patients with HR-positive, HER2-negative MBC is HT combined with cyclin-dependent kinase 4/6 (CDK4/6) inhibitors. The best HT option is determined by different factors, such as menopausal status, previous HT, and comorbidities. Clinical benefit from HT predicts patient survival and the possible benefit of subsequent HT [6]. It is estimated that approximately one-third of HR-positive patients present primary resistance to HT, the vast majority of whom develop secondary resistance. However, these same patients may respond to other types of HT. Therefore, the second-, third-, and fourth-line HT is administered sequentially, as resistance develops and the disease progresses [6, 7]. Despite the fact that new parameters defining hormone sensitivity have been described [7], hormone sensitivity and resistance criteria based on clinical criteria and agreed by expert consensus have now been adopted, even in the absence of definitive biological data other than the expression or non-expression of HR-positive, HER2-negative receptors [8]. A full definition of these criteria would help optimize health-care processes and improve decision-making in the future.

Consensus documents are valuable tools that assist in the decision-making process for establishing clinical strategies and optimize the delivery of health services [9]. The preparation of this consensus document has been supported by the GEICAM Breast Cancer Research Group with the aim of developing recommendations on criteria for hormone sensitivity and resistance in HER2-negative luminal MBC and facilitating clinical decision-making.

## Materials and methods

This consensus document was generated using a modification of the RAND/UCLA methodology [10]. The project phases were as follows. (1) Definition of the project and identification of issues of interest: objectives, methodology, and clinical questions to be answered in the consensus document were established. In addition, the working group was formed by a coordinator and a consensus group composed of seven oncologists, all experts in breast cancer who constituted the GEICAM working group on luminal disease between 2015 and 2018. (2) Non-exhaustive systematic review of the literature: we carried out a review of the scientific evidence from studies published between January 2000 and April 2016, in the following databases: PubMed, Guía Salud, Cochrane, Trip database, and Uptodate. The clinical questions were reformulated into questions using the PICO format (population/patients, intervention, comparators, outcomes) [11]. This search was complemented with a manual search of references by the consensus group aimed at completing the evidence with articles that could be helpful for answering the questions raised. (3) Analysis and synthesis of the scientific evidence: the members of the consensus group were responsible for systematically reviewing the available scientific evidence. After a critical reading of the full text of the selected studies, they prepared a summary using a standardized format, including tables and text to describe the methodology, the results, and the quality of each study. The reasons for exclusion of articles not included in the selection were listed. Scientific evidence and classification of the recommendations were evaluated according to the Oxford Center for Evidence-Based Medicine (CEBM) system [12]. (4) Preparation of recommendations: the consensus group drew up answers to the clinical questions from the scientific evidence and expert clinical judgment. All responses included the appropriate reasoning and proposed conclusion(s) and recommendation(s). (5) External evaluation: the recommendations/conclusions drawn up by the consensus group underwent a process of external validation using a two-round Delphi technique, with a panel of 64 medical oncologists specializing in breast cancer representing the validation group. In each round, the members of the validation group expressed their degree of agreement or disagreement with the recommendations/conclusions using an online questionnaire with a Likert scale of 1–4 (1 = strongly disagree; 4 = strongly agree). The recommendations with a level of agreement ≥ 80% [sum of scores of 3 (agree) and 4 (strongly agree)] were accepted. In total, all 32 of the 32 recommendations/conclusions presented in the first round were accepted (approval rate of 100%).

## Results

### 1. Definition of metastatic disease or locoregional recurrence not susceptible to local curative treatment

The luminal subtype is characterized by overexpression of estrogen receptors (ER) and/or progesterone receptors (PR). HR status is determined using immunohistochemical techniques on tumor biopsies. The tumor is considered HR positive if HR expression is detected in at least 1% of the nuclei of the invasive tumor cells. Staining in ≥ 10% of the nuclei indicates unambiguous positivity for the indication of HT; if staining is observed in 1–10% of the nuclei, the pros and cons of HT must be weighed up. Tumors without ER expression but with positive PR expression should be considered RH-positive tumors that may benefit from HT [13, 14].

#### Is it necessary to determine the intrinsic luminal subtype using gene expression platforms for therapeutic decision-making in luminal MBC?

The clinical heterogeneity of breast cancer patients with positive HR is caused by the different gene expression profiles that define luminal subtypes A and B [15]. With regard to the classification of the tumor using gene platforms, such as PAM50, several studies have been performed to validate the results obtained with several variables of clinical interest. These studies have shown that it is possible to identify all intrinsic genomic subtypes in patients with HR-positive, HER2-negative tumors [16, 17]. This genomic information should have greater prognostic value than conventional clinical and pathological data [17, 18], and genomic profiles are more effective than immunohistochemical techniques for identifying patients with luminal MBC that will respond to HT [PFS in patients with luminal subtype: 11.0–16.9 months; PFS in patients with non-luminal subtype: 4.1–4.7 months] [17]. The latest recommendations of the American Society of Clinical Oncology (ASCO) on HT in luminal MBC specify that intrinsic genomic subtypes have been associated with prognosis, but their usefulness for selecting the most effective treatment has not yet been demonstrated. Thus, the determination of intrinsic subtypes using genomic profiles is considered experimental and should be reserved for the selection of patients in the clinical trial setting [19].

#### Can luminal A and B MBC subtypes be differentiated using immunohistochemical techniques?

With the aim of associating genomic data with clinical and pathological characteristics, several studies have been conducted to establish differences in the receptor expression and proliferative status of tumors among the different luminal genomic subtypes using immunohistochemical techniques [20, 21]. Ki-67 was the marker selected to determine proliferation, defining positivity at a cutoff point of 14–20% [20, 22, 23]. The luminal subtype A would be defined by an expression below this cutoff point. PR levels have also been proposed as markers that might help discriminate between the two subtypes, with a cutoff point set at 20%. Thus, tumors with more than 20% of PR-positive cells would be considered luminal A. Published evidence indicates that tumors with immunohistochemical luminal subtype A treated with HT may have a better response rate (RR), PFS, and overall survival (OS) post-relapse [16, 24–26] and appear to be less sensitive to treatment with chemotherapy [27] than luminal subtype B tumors.

#### What are the criteria for defining menopause?

It is essential that criteria for determining menopause are defined in patients with luminal breast cancer because, bearing in mind the mechanism of action of aromatase inhibitors (AIs), only patients that meet strict post-menopausal criteria will be candidates to receive these agents, in both early and metastatic disease [28]. The problem lies in the fact that the definition of menopause varies among the different clinical trials and research groups [29]. Menopause is a steep and permanent drop in ovarian estrogen synthesis, although this definition is not supported by any fixed criteria, particularly with regard to age or months of amenorrhea [30]. Several definitions of menopause are listed in Table 1.

**Table 1:** Definitions of menopause from clinical practice guidelines

NCCN [100]	Prior bilateral oophorectomy ≥ 60 years < 60 years, and amenorrhea ≥ 12 months in the absence of chemotherapy, tamoxifen, toremifene, or Ovarian suppression, and levels of FSH and estradiol within postmenopausal ranges In case of treatment with tamoxifen or toremifene and age < 60 years, FSH and estradiol plasma levels must be within postmenopausal ranges^a^
ASCO [19]	Lack of menstruation in the past 12 months in the absence of chemotherapy Oophorectomy, or Ovarian suppression with LHRH agonists
NICE [101]	> 45 years with no menstruation in the last 12 months and not taking hormonal contraception, or > 45 years with hysterectomy and menopausal symptoms^b^

*ASCO* American Society of Clinical Oncology, *FSH* follicle-stimulating hormone, *LHRH* luteinizing hormone-releasing hormone, *NCCN* National Comprehensive Cancer Network, *NICE* National Institute for Health and Care Excellence

^a^A postmenopausal status cannot be assigned to women who are receiving LHRH antagonists

^b^Only FSH levels will be analyzed to diagnose menopause when patients are between 40 and 45 years of age and present menopausal symptoms, including changes in menstrual cycle or age < 40 years, suspicion of menopause, not receiving treatment with combination hormonal contraception (estrogen and progestin) or high doses of progestin

#### Should metastatic disease be biopsied at the time of its appearance and should the result guide the therapeutic decision?

Traditionally, the levels of various biological markers in the primary tumor have guided systemic treatment in metastatic disease, but sometimes markers in metastatic disease can differ with respect to the primary tumor findings. A lesion suspected of being metastatic breast cancer might be benign or it might be a second malignant tumor, thus altering prognosis and treatment. Various prospective studies show that in 4–10% of cases, lesions suspected of being metastatic correspond in reality to healthy tissue, benign lesions, or a second malignant tumor, underlining the importance of biopsy for confirming the existence of breast cancer metastasis, particularly at the time of initial relapse. An analysis of the ConvertHER study using PAM50 confirmed changes in the molecular subtype between the primary tumor and the metastasis [31, 32]. A meta-analysis [33] of data from individual patients from two of these studies (DESTINY and BRITS) showed rates of conversion between the primary tumor and the metastasis of 6%, 13%, and 31% for HER2, ER, and PR, respectively. The treatment plan was modified in 8–14% of cases after biopsy. These results are similar to those obtained in the previous study. Most clinical guidelines recommend a biopsy of metastatic lesions (one or more) at the time of presentation of metastatic disease, to confirm the nature of the metastases and to re-evaluate HR and HER2 status [19, 34–36]. Caution should be exercised with the results obtained from tissue biopsies in which technical difficulties are a likelihood, such as bone biopsy [8, 19, 34].

Table 2 shows the recommendations and conclusions of the expert group with regard to this topic.

**Table 2:** Recommendations and conclusions on the definition of metastatic or recurrent locoregional luminal disease not susceptible to curative local treatment

	LA (%)	LE/RD
HR-positive tumors are those with ≥ 1% expression of ER and/or PR in the nuclei of breast cancer tumor cells [13, 14]	90	1
Determination of intrinsic luminal subtypes using gene expression should not be the only factor considered in decision-making in luminal MBC. Decisions on the treatment of luminal MBC (HR-positive, HER2-negative) have to be made taking into account biomarker results (ER, PR, HER2) from immunohistochemistry and ISH (expert opinion)	92	5/D
Menopause is defined based on the following criteria: Age ≥ 60 years or Bilateral oophorectomy or Age < 60 years and ≥ 12 months of amenorrhea in the absence of chemotherapy, tamoxifen, toremifene, or ovarian suppression, with levels of FSH and estradiol within postmenopausal ranges In case of treatment with tamoxifen or toremifene and age < 60 years, FSH and estradiol plasma levels must be within postmenopausal ranges* [28]	97	5/D
Premenopausal patients who have received chemotherapy may present anovulation/amenorrhea, without implying a loss of ovarian function. Therefore, FSH and estradiol levels persistently within menopausal ranges with close monitoring for a period of at least 2 years must be demonstrated to assign a post-menopausal status (expert opinion)	95	5/D
A biopsy (obtaining a tissue sample) of metastatic disease, if easily accessible, must be performed to confirm the diagnosis, especially at the beginning of metastatic disease [34–36]	99	1/B
Whenever clinically possible and technically appropriate, the study of biomarkers (ER, PR, and HER2) in metastatic disease is recommended (at least at the beginning of metastatic disease), as these markers can change in disease progression with respect to the primary tumor, so the most appropriate systemic treatment can be selected (although insufficient evidence is available to suggest that re-evaluation of biomarkers improves prognosis). This procedure should always be carried out if the biomarker status of the primary tumor is unknown and there is no access to it [34–36]	99	1/B
When discrepancies are found between ER, PR or HER2 biomarkers in the primary tumor and the metastases, the ER, PR and HER2 status of the metastasis should be used to guide systemic treatment, provided that it is compatible with the setting/clinical judgment. In this case, serial biopsies from various locations are recommended for a better assessment of biomarker progress [35, 37]	88	5/D

*ER* estrogen receptor, *ESR1* estrogen receptor 1, *HER2* human epidermal growth factor receptor 2, *HR* hormone receptor, *LA* level of agreement, *LE* level of evidence, *MBC* metastatic breast cancer, *NSAI* non-steroidal aromatase inhibitors, *PR* progesterone receptor, *RD* recommendation degree according to Oxford

### 2. Definition of hormone sensitivity and hormone resistance in metastatic luminal disease

#### What variables can be used to set the definition of hormone sensitivity and hormone resistance in luminal MBC?

It is of the utmost importance that variables determining hormone resistance are identified, both for making decisions in clinical practice and for interpreting clinical trials [8, 19, 37–39]. In this setting, changes in HR expression in metastatic cells must be evaluated, although there is no evidence that these changes are predictive of response to any type of therapy [8, 19, 37, 40, 41]. Changes in HER2 expression in metastasis with respect to the primary tumor must also be taken into account. Several randomized clinical trials have demonstrated that the combination of HT and anti-HER2 agents can overcome this resistance, at least in part [42, 43]. Vascular endothelial growth factor (VEGF) has also been linked to HT resistance in retrospective studies, although this observation was not validated in prospective studies conducted with bevacizumab, which produced controversial results and failed to prevent the emergence of resistance [44, 45]. Other markers, such as Ki-67, p53, BCL-2, PI3K, CCDN1, p16, or multigene platforms, have not been useful in therapeutic decision-making [19, 46, 47]. For this reason, these markers must only be considered in the context of clinical trials [19, 46, 47]. Other markers of resistance are estrogen receptor ESR1 gene mutations, which are present in 30% of metastases in patients who have received HT, but normally absent in the primary tumor [48]. Although these mutations can plausibly be identified, their ability to predict a good response to HT must be validated in prospective studies [48].

Two different scenarios must be considered when establishing the possible definition of primary or secondary hormone resistance. One would be time-dependent, defining relapse after adjuvant treatment, and the other would consider time to progression during the treatment of metastatic disease. If relapse occurs within 2 years after beginning adjuvant HT, or during the first 6 months of first-line HT, experts consider that resistance or insensitivity to HT is primary or intrinsic. In turn, when the diagnosis of relapse occurs more than 2 years after starting adjuvant HT, but within 1 year of completing adjuvant therapy, or if progression occurs later than 6 months after starting HT for metastatic disease, experts consider that resistance is acquired or secondary [19]. Regardless of the time periods, if relapse occurs during adjuvant HT, the disease is resistant to that specific hormonal strategy [49]. The same occurs if metastatic cancer progresses within a period of less than 1 month after discontinuation of such treatment [19, 38, 39, 50]. Table 3 shows some of the definitions used in clinical practice guidelines.

**Table 3:** Main definitions of primary and secondary hormone resistance

Reference	Primary hormone resistance	Secondary hormone resistance	Level of evidence
European Consensus guidelines [34]	Relapse occurred during the first 2 years of adjuvant HT, or Progressive disease in the first 6 months of HT in MBC	Relapse occurred after the first 2 years of adjuvant HT or in the first year after completion of adjuvant HT Progressive disease occurring after the first 6 months of HT in MBC	5
GINECO study [57]	Relapse during adjuvant HT or in the first 6 months after completion of adjuvant HT Progressive disease in the first 6 months of HT in MBC	Relapse occurring later than 6 months after completion of adjuvant HT, or Progression after 6 months of starting HT in MBC	2
Review articles: [102, 103]	Progressive disease without initial response to treatment	Progressive disease after initial response to treatment	5

#### Is there evidence for suggesting that a combination of several hormonal drugs can reverse hormone resistance and increase effectiveness compared to single-agent hormone therapy?

Two phase III clinical trials exploring this issue in postmenopausal women in first-line treatment for MBC, using a combination of anastrozole and fulvestrant, produced diverging results [1 positive: SWOG 0226 [51] (HR (hazard ratio): 0.81; 95% confidence interval: 0.65–1.00), and 1 negative: FACT [52] (HR: 1.0; 95% CI: 0.76–1.32)], although when a subgroup analysis of the SWOG cohort was conducted, there was a significant benefit in OS in patients who had never received adjuvant HT (HR: 0.74; 95% CI: 0.56–0.98) [51]. The combined meta-analysis of both studies found a marginal but non-significant benefit in PFS (HR: 0.88; 95% CI: 0.72–1.09) and OS (HR: 0.88; 95% CI: 0.72–1.08) [53].

Another study in postmenopausal women who had progressed with a non-steroidal aromatase inhibitor (NSAI) did not find any kind of advantage with the combination of anastrozole and fulvestrant in comparison with single-agent fulvestrant or exemestane [54]. In premenopausal women, the combination of ovarian suppression with tamoxifen in first line was superior to ovarian suppression in terms of OS (HR: 0.78; 95% CI: 0.63–0.96) and PFS (HR: 0.70; 95% CI: 0.58–0.85) [55].

The international guidelines of the European School of Oncology–European Society of Medical Oncology (ESO–ESMO) and ASCO recommend the use of sequential single-agent HT in postmenopausal patients [19, 34] and only recommend the combination of anastrozole and fulvestrant in patients who have not received any adjuvant treatment [19]. In the case of premenopausal patients, a combination of ovarian suppression with tamoxifen is recommended in first line, and in subsequent lines, ovarian suppression should be maintained, and patients should be treated in the same way as postmenopausal women [19, 34].

#### Is there evidence for suggesting that HT combined with chemotherapy can reverse hormone resistance and increase effectiveness compared to single-agent hormone therapy?

The analysis of some studies suggest that the sequential use of each type of treatment is equivalent to their combination, and data in the adjuvant setting even suggest that combined use may be less effective than sequential use [56]. However, the luminal subtype has not been specifically investigated in any recent study. Therefore, international clinical guidelines do not recommend the combination of HT with chemotherapy [19, 34].

#### Is there evidence for supporting the use of HT combined with targeted therapies instead of single-agent hormone therapy to reverse hormone resistance and increase effectiveness in HR-positive, HER2-negative MBC?

One of the possible mechanisms of resistance to HT is activation of the cellular pathways that might interfere with inhibition of the hormone-dependent pathway. One of these pathways is PI3K. Several studies have been published on the mTOR inhibitors, including everolimus and tamoxifen [time to progression (TTP) HR: 0.54; 95% CI: 0.36–0.81] [57] and everolimus and exemestane (OS: HR: 0.89; 95% CI: 0.73–1.10) [58]. As such, everolimus has been approved for use in combination with exemestane after failure of an NSAI, as reflected in international clinical guidelines [19, 34]. Tensirolimus in combination with fulvestrant (PFS: HR: 0.61; 95% CI: 0.40–0.92) [59] showed a significant increase in PFS. A phase III study evaluated the effect of a combination of tensirolimus and letrozole in first-line therapy, and did not find a benefit in PFS for the combination (HR: 0.90; 95% CI: 0.76–1.07) [60]. The data on PI3K inhibitors are still very preliminary. The SOLAR-1 study has shown an increase in PFS in second-line treatment in patients with tumors with a PI3K mutation (HR: 0.65; 95% CI: 0.50–0.85) [61].

Two studies in first line with bevacizumab in combination with letrozole produced diverging results, one being negative for PFS (HR: 0.83; 95% CI: 0.65–1.06) [45] and the other positive (HR: 0.75; 95% CI: 0.59–0.96) [44]. For this reason, its use has not been recommended in clinical guidelines [62].

The use of anti-EGFR inhibitors (HER1), such as gefitinib, or anti-HER1/2, such as lapatinib, in patients with HR-positive, HER2-negative MBC has not proved effective and is not recommended [63–65].

Three cyclin-D-dependent kinase (CDK)4/6 inhibitors, ribociclib, abemaciclib, and palbociclib, have been studied. In the case of palbociclib, data from two studies (a randomized phase II study [66] and a phase III study [67] in which palbociclib was combined with letrozole in first-line therapy in postmenopausal women) have been published. Both studies showed an increase in PFS with HT alone. There are also data on second-line treatment in combination with fulvestrant, which show an increase in PFS compared to HT alone in both postmenopausal and premenopausal women, associated in the latter with ovarian suppression with luteinizing hormone-releasing hormone (LHRH) analogues (HR: 0.49; 95% CI: 0.32–0.75) and HR: 0.50; 95% CI: 0.33–0.76], respectively) [38, 68]. In the case of ribociclib, data have been published on a phase III study in first line in postmenopausal women, in which it was combined with letrozole, showing an increase in PFS compared to HT alone (HR: 0.56; 95% CI: 0.43–0.72) [69]. In the case of abemaciclib, data have been published from two studies: a phase III study, in which abemaciclib was combined with an NSAI, anastrozole, or letrozole, in first line [70] and another phase III study in which it was combined with fulvestrant in second line [71]; in both cases an increase in PFS was found compared with HT alone (HR: 0.54; 95% CI: 0.41–0.72 and HR: 0.55; 95% CI: 0.45–0.68), respectively). Data have also been presented from a phase II study of abemaciclib monotherapy in patients polytreated with HT and chemotherapy, showing a response rate of up to 17% [72].

Table 4 shows the recommendations of the expert group with regard to hormone resistance and hormone sensitivity.

**Table 4:** Recommendations and conclusions on the definition of hormone sensitivity-hormone resistance in metastatic luminal disease

	LA (%)	LE/RD
Negativization (< 1%) of hormone receptors in metastatic cells should be considered as hormone resistance, so ER, PR and HER2 of a new metastasis should be determined whenever possible before making therapeutic decisions (expert opinion)	95	5
A high level of hormone receptors makes hormone sensitivity more likely. However, there is no accurate cutoff point or score that indicates hormone resistance, so any tumor with ER ≥ 1% can theoretically be hormone sensitive (expert opinion)	93	5
The only unequivocal criterion of hormone resistance is progression during a hormonal intervention. Progressive disease during the first 6 months of HT for MB or during the first 2 years of (neo-)adjuvant HT is evidence of resistance to hormone therapy, so the line of treatment must be changed [19, 50]	97	2
Intracellular signaling markers and ER mutations (ESR1) should not be taken into account when deciding to use HT outside the setting of a clinical trial [19, 37]	93	2/B
In the context of disease relapse after adjuvant treatment, progression occurring during the first 2 years of adjuvant treatment can be considered as primary or refractory hormone resistance [34]	97	5
In the context of MBC, progression in the first 6 months of HT can be considered primary hormone resistance [34]	97	5
Insufficient evidence was found to answer this question. However, in the context of relapse during or after adjuvant treatment, relapse occurring later than 2 years after starting treatment and within 1 year after completion could be considered secondary hormone resistance [34]	95	5
In the context of metastatic disease, progressive disease after an initial benefit with HT within the first 6 months of treatment is considered secondary hormone resistance [34]	92	5
In postmenopausal women, there is insufficient evidence to recommend the combination of two hormone agents simultaneously. Thus, HT in sequential monotherapy is recommended [19, 34]	97	1/A
In the case of premenopausal women, a combination of ovarian suppression with tamoxifen or AI in first line is recommended [19, 34]	95	1/B
In premenopausal women, ovarian suppression should be maintained and sequential HT should be added as in postmenopausal women in second line and in subsequent lines [19, 34]	93	3/C
In patients with HR-positive, HER2-negative advanced breast cancer, the use of a combination of TH and chemotherapy is recommended. Sequential administration is recommended, with HT being the preferred option in first line and in successive lines until the emergence of resistance. The use of chemotherapy as a first option is preferable in a critical situation of visceral disease requiring a quick response [19, 34, 56]	99	2/C
In postmenopausal patients, cyclin-dependent kinase inhibitors ribociclib and palbociclib in combination with an NSAI are recommended as a treatment option in first-line HT. Palbociclib is also recommended in second line in combination with fulvestrant after failure on first-line therapy (palbociclib is also indicated in premenopausal* patients in combination with an NSAI or fulvestrant with an LHRH analog) [38, 66–69, 74]	97	1/A
In postmenopausal patients, the combination of exemestane with everolimus is recommended in the absence of symptomatic visceral disease after failure of an NSAI. The use of mTOR inhibitors is not recommended in patients who have not previously received an NSAI or in cases of MBC de novo or those who have relapsed after more than 1 year after the end of adjuvant treatment [19, 34]	97	2/B
The use of antiangiogenic drugs in combination with HT is not recommended [19, 34]	89	2/B
The use of HER1/2 inhibitors in combination with HT in HR-positive, HER2-negative MBC is not recommended [63–65]	95	2/B

*ER* estrogen receptor, *ESR1* estrogen receptor 1, *HER2* human epidermal growth factor receptor 2, *HT* Hormone therapy, *LA* level of agreement, *LE* level of evidence, *MBC* metastatic breast cancer, *mTOR* mammalian target of rapamycin, *NSAI* non-steroidal aromatase inhibitors, *PR* progesterone receptor, *RD* recommendation degree according to Oxford

*Currently, ribociclib and abemaciclib are also indicated in premenopausal patients. Palbociclib is the only cyclin-dependent kinase inhibitor mentioned because ribociclib and abemaciclib had not still the indication in this subgroup of patients when the external validation using a two-round Delphi technique was done

### 3. Therapeutic decision-making

#### Can the menopausal status influence the selection of first-line treatment? Should ovarian function be suppressed in first-line treatment?

The selection of the optimal HT in patients with HR-positive, HER2-negative MBC should be guided by menopausal status, previously administered HT, and the patient’s comorbidities [19, 73]. Very few clinical trials have included premenopausal women in their designs, especially in first-line strategies [74]. The choice of treatment in first line depends on the adjuvant treatment, which may be chemical or surgical castration [19]. Studies comparing the effectiveness of tamoxifen and oophorectomy in this type of patients found no significant differences for the various clinical parameters under study [75, 76]. LHRH analogs with or without tamoxifen had similar results to oophorectomy [77]. The combination of LHRH analogs and tamoxifen was superior to tamoxifen in terms of response rate, TTP, and OS [78].

#### What are the criteria for tumor “aggressiveness” in luminal MBC that would indicate the need for chemotherapy?

Several studies have demonstrated similar rates of disease control and OS (HR: 0.94; 95% CI: 0.79–1.12) between HT and chemotherapy [79]. Although chemotherapy can achieve a greater number of responses, the toxicity profile favors HT. In addition, the presence of visceral disease (non-life-threatening) has not been shown to reduce the benefit of HT. Numerous reviews highlight HT as the treatment of choice in first line and in subsequent lines of treatment in HR-positive, HER2-negative MBC, reserving chemotherapy for cases with imminent failure of a vital organ [56, 80], symptomatic visceral disease, and/or rapid growth [56, 80, 81], or HT resistance, including previous combinations of HT and targeted therapies, such as mTOR inhibitors or cyclin-dependent kinase inhibitors, the aim of which is to delay or reverse the development of this endocrine resistance [81, 82]. These results have prompted clinical practice guidelines such as ASCO to consider chemotherapy as an appropriate initial treatment in patients with imminent life-threatening disease, in whom the time to response can be critical. It may also be considered as a first option in patients with low HR levels in whom HT may be less effective [19]. The ESO–ESMO clinical guidelines for MBC also assert the recommendation to reserve chemotherapy for cases with rapidly progressive disease or confirmed endocrine resistance. In other situations, HT is still the first option, even in the presence of visceral disease [34]. The National Breast and Ovarian Cancer Center (NBOCC) guidelines also recommend using chemotherapy only in cases of rapidly progressing visceral disease [83]. The Canadian guidelines offer alternatives in postmenopausal women with HR-positive, HER2-negative MBC who relapse or progress after treatment with an NSAI, proposing other hormone treatments or a combination of everolimus and exemestane in patients with endocrine resistance and slow progression. Chemotherapy is limited to patients with resistance to HT and symptomatic visceral disease [78]. The Spanish Society of Medical Oncology (SEOM) guidelines for MBC published in 2018 specify that chemotherapy should be standard treatment in patients who are refractory to HT [84].

#### Are serum biomarkers useful?

A series of serum biomarkers has emerged in recent years, including CA15.3, CEA, CA 27.29, circulating tumor cells (CTC), and circulating erbB-2 extracellular domain that could help determine resistance to hormonal interventions. Some prospective studies have shown that the combination of several of these serum markers, such as CEA and CA 15.3 [85, 86], or CTC and CA 27.29 [87], is more sensitive than using them singly in the diagnosis of metastatic relapse. However, randomized phase III studies have not shown any survival benefit from the serial determination of serum markers [88], nor is there any evidence that initiating or changing treatment on the basis of serum markers or CTC confers an improvement in health status, survival, or quality of life [19, 34, 37, 88]. Three expert consensus articles also agree that no well-designed studies are available to evaluate the clinical utility of the markers [19, 34, 37]. Nevertheless, this is the only method for monitoring unevaluable metastatic disease [19, 34, 37, 85, 86].

Table 5 shows the recommendations of the expert group with regard to therapeutic decision-making.

**Table 5:** Recommendations on therapeutic decision-making

	LA (%)	LE/RD
Menopausal status should not be taken into account when deciding whether to use chemotherapy or HT in patients with ER-positive, HER2-negative MBC [79]	89	5/D
Menopausal status should be taken into account when selecting HT in patients with ER-positive, HER2-negative disease [19, 73]	90	5/D
In premenopausal patients with ER-positive MBC, ovarian ablation in combination with another HT should be recommended. In patients who have never received HT or who relapse more than 12 months after completing adjuvant HT, combined ovarian ablation or suppression should be recommended [75, 76]	96	2/B
In premenopausal patients with ER-positive MBC who have never received HT or who relapse after 12 months since completing adjuvant HT, ovarian suppression or ablation should be recommended in combination with tamoxifen or AI ± cyclin inhibitors as first-line treatment, provided that there is no visceral crisis [55]	97	1/A
In premenopausal patients with ER-positive MBC who relapse during adjuvant HT or in the 12 months after completion of adjuvant HT, ovarian ablation could be combined with an AI or fulvestrant, as in postmenopausal patients, although the evidence is still inadequate [78]	93	3/C
Suggested criteria for tumor “aggressiveness” in luminal MBC that would indicate the need for treatment with chemotherapy are as follows: Rapidly progressing or very symptomatic disease Disease compromising a vital organ or imminent risk to life Disease with demonstrated endocrine resistance, even after previous treatment with targeted therapies (mTOR or cyclin inhibitors, for example) casting doubt on the possible response to HT (expert opinion)	99	5/D
Serum markers may not be used as the sole criterion for defining progression (resistance) or for starting, stopping or changing HT. However, progressive and sustained elevation (two determinations more than 1 month apart) of CA 15. 3 and/or CEA could be useful in unmeasurable disease to raise suspicion of progression (resistance) and to request imaging tests to confirm progression (evaluation of PET/CT) (expert opinion)	97	5/D
In the event of a sustained elevation of serum marker CA15.3 and/or CEA during HT, imaging studies should be requested to document any progression/hormone resistance. At this stage, PET/CT is more sensitive than conventional methods, especially in the evaluation of pathological axillary, supraclavicular, internal mammary chain, and mediastinal lymphadenopathies [56, 85]	90	3/C
CTC and circulating c-erbB-2 extracellular domain should not be used as the sole criterion to start, stop or change HT [19, 34, 37, 88]	99	2/C

*AI* aromatase inhibitor, *CTC* circulating tumor cell, *ER* estrogen receptor, *HER2* human epidermal growth factor receptor 2, *HT* hormone therapy, *LE* level of evidence, *LA* level of agreement, *MBC* metastatic breast cancer, *mTOR* mammalian target of rapamycin, *PET* positron emission tomography, *RD* recommendation degree according to Oxford

## Discussion

Consensus documents prepared by independent committees are a valuable tool for decision-making in areas in which evidence is limited or could not be combined to establish clinical strategies or improve efficiency in the delivery of health services [9]. The preparation of this consensus document has been sponsored by the GEICAM Breast Cancer Research Group, and is the product of the dedicated involvement of the members of the GEICAM Luminal Disease Working Group. This consensus was developed following a robust, recognized, and rigorous methodology, but a number of limitations inherent to documents of this type must be taken into account. In the first place, the review of the literature, while systematic, was not exhaustive, and only the most current publications were included. Furthermore, after the initial literature review, the development of recommendations and consensus took longer than expected, which is why, after they were completed, we had to perform an additional search to update specific themes to take into account recent advances in the treatment of luminal MBC. The following is a summary of the main results of this additional review.

With respect to the combination of everolimus with HT, results have been published showing that the combination of everolimus with fulvestrant in second-line therapy increases PFS from 5.1 to 10.3 months (HR: 0.61; 95% CI: 0.40–0.92) [89], results that are in line with data from the phase III study of everolimus plus exemestane [90]. With regard to PI3K inhibitors (selective or pan-PI3K inhibitors), the results of phase III trials of buparlisib [91, 92] and taselisib [93], both in combination with fulvestrant, have recently been published. Toward the end of 2018, data were presented on alpelisib (a selective inhibitor of the PI3K alpha subunit) in combination with fulvestrant showing increased PFS in patients with PIK3CA mutations [61]. While the first two showed positive but clinically non-significant results, the selective inhibitor alpelisib did yield data on statistically significant efficacy and clinically manageable safety in the second-line treatment of patients with ER+/HER2−/PI3CA-mutated breast cancer. With regard to CDK4/6 inhibitors, the results of phase III studies of ribociclib combined with tamoxifen and LHRH analogs in first line in premenopausal patients [94] and with fulvestrant in first and second line in postmenopausal patients [95] have been published, showing the same benefit in PFS. These results give greater coherence to the use of CDK4/6 inhibitors in combination with HT in the first- or second-line treatment of luminal MBC. Finally, data were presented at ASCO 2019 on the impact on OS of ribociclib combined with HT in premenopausal patients who, for the first time, showed an increase in OS with the combination of HT and CDK4/6 inhibitors [96], and later in ESMO 2019 the benefit in OS has been presented in other two trials, one for the combination of ribociclib with fulvestrant in first and second line in postmenopausal women (MONALEESA3) [97] and the other for abemaciclib in combination with fulvestrant in second line [98].

The classification of luminal subtypes A and B is based on gene expression and their correlation with immunohistochemistry is not complete. There is no evidence to suggest that the subclassification of luminal type A or B using immunohistochemical techniques, in the absence of other factors, should influence therapeutic decision-making. A meta-analysis which included the analysis of 39 studies found that the conversion rates of receptors in metastasis of advanced breast cancer patients are high. It is recommended that biopsies are performed to confirm the status of these receptors in metastasis, although more prospective studies would need to be conducted to determine whether this conversion has some type of impact on the effectiveness of treatment in terms of survival [99].

Medical oncology is a field in which new data are constantly emerging. These updates oblige us to respond to questions that arise from the new evidence, including, for example, the diagnostic role of new technologies, such as genomic platforms and liquid biopsy. Other areas of therapeutic interest might be determining suitable patient profiles for biologics and combination therapies (HT with biologics), as well as their sequence. Other issues might address those profiles in which chemotherapy will be more effective or the duration of administration of antiresorptive therapies. With regard to follow-up, questions are being raised on the aggressiveness of tumors that have developed resistance to cyclin-dependent kinase inhibitors, the role of fulvestrant in first line to avoid the appearance of ESR1 mutations, and quality of life issues. Other questions to be resolved surround the primary objectives of clinical trials and the role of combined therapies in advanced luminal cancer. Answers to these and other questions should become available from future studies to be able to continue offering improvements in the treatment of patients with advanced luminal breast cancer.
